# Assessing the predictive validity of pre-admission criteria on dental students’ academic performance: a cross-sectional study

**DOI:** 10.1186/s12903-023-03839-6

**Published:** 2024-01-16

**Authors:** Sultan A. Almalki, AlBandary H. AlJameel, Ziyad Alghomlas, Tameem Alothman, Fawaz Alhajri

**Affiliations:** 1 Department of Preventive Dental Sciences, College of Dentistry, Prince Sattam Bin AbdulAziz University, 11942 Al-Kharj, Saudi Arabia; 2 Department of Periodontics and Community Dentistry, College of Dentistry, King Saud University, 11545 Riyadh, Saudi Arabia; 3 College of Dentistry, Prince Sattam Bin AbdulAziz University, 11942 Al-Kharj, Saudi Arabia

**Keywords:** Academic performance, Academic success, Students, Schools, Dental education, School admission criteria, Selection, Undergraduate, Pre-admission criteria

## Abstract

**Background:**

This study examines the multifaceted factors influencing academic performance among dental students in Saudi Arabia, exploring the complex relationships between demographic, educational, and psychological variables and students' Grade Point Averages (GPAs) to enhance global dental education admission strategies.

**Methods:**

Data on demographics, academic performance indicators (including GPA, high school grades, test scores), accommodation type, parental education, suspension history, and English proficiency were collected through an English-language electronic questionnaire from 900 dental students across multiple institutions in Riyadh. The study employed Pearson’s correlation analysis to examine relationships between GPA and various academic and demographic factors. A detailed regression analysis, using a stepwise selection based on Akaike’s information criteria (AIC), identified significant GPA predictors and evaluated the average marginal effects of pre-admission variables on GPA, alongside rigorous diagnostic checks to validate the model’s robustness.

**Results:**

The study revealed a complex interplay of factors impacting GPA among dental students. High school grades, achievement, and aptitude test scores showed moderate positive correlations with GPA, while negative correlations were observed with age and number of times suspended. Regression analysis highlighted age, marital status, high school grades, and test scores as key predictors, with complex interaction effects demonstrating the layered influences of these factors. For instance, the negative impact of age on GPA was moderated by high school grade and aptitude test score. The analysis also highlighted the significant marginal effects of various pre-admission variables on GPA, such as the negative average impact of age and the positive impact of high school grades.

**Conclusions:**

This study provides valuable insights into the multifaceted determinants of academic success in dental education. Our findings underscore the significant roles of high school grades, achievement, and aptitude test scores, as well as the nuanced influence of age and marital status on GPA. These results advocate for a holistic approach in evaluating candidates for dental programs and have broader implications for global dental education, emphasizing the need for comprehensive admission strategies.

## Background

The selection process for dental school admission is integral in shaping the caliber of future dental professionals globally. This aspect assumes heightened significance in the context of Saudi Arabia, where a surge in applicants and distinct educational characteristics underscore a critical research gap. There is a conspicuous absence of in-depth studies evaluating how pre-admission criteria forecast the academic achievements of dental students, particularly within the Saudi Arabian milieu. Addressing this gap is imperative, given the dynamic nature of dental education that demands a blend of cognitive competencies and non-cognitive skills [1–4].

Saudi Arabian dental colleges employ a multifaceted admissions approach, encompassing aptitude and achievement tests, high school grades, and often, personal interviews. Prior to entering dental programs, students must complete a preparatory year focusing on English and basic sciences. Each of these components plays a role in assessing a student's potential for success in dental education. However, the precise impact of these criteria on the academic performance of dental students in Saudi Arabia remains largely unexplored.

Our research addresses this lacuna by hypothesizing that, alongside cognitive abilities, non-cognitive skills significantly influence dental students' Grade Point Averages (GPAs) in Saudi Arabia. This hypothesis stems from an increasing acknowledgment of the importance of psychomotor, interpersonal, and communication skills in dental education for achieving clinical excellence [5–8]. While the role of achievement tests as performance indicators is well-established in medical education [9], their relevance in dental education, especially in Saudi Arabia, requires further elucidation.

The study's findings revealed key predictors of academic success, such as the significant impact of high school grades, age, living arrangements, suspension history, and English proficiency levels on students' GPAs. These insights hold crucial implications for admission strategies, suggesting the need for a more comprehensive evaluation of applicants that extends beyond traditional academic metrics.

This study contributes to a nuanced understanding of the factors influencing dental students' academic performance, thereby informing the development of admission standards that better predict student success. It underscores the necessity of incorporating diverse factors, including non-academic elements, in the admission processes. Such an approach promises to enhance the effectiveness of dental education in Saudi Arabia and potentially globally, aligning admission practices with the multifaceted requirements of contemporary dental education.

## Methods

This cross-sectional study targeted all undergraduate dental students in Riyadh, Saudi Arabia, from eight colleges, including five governmental and three private institutions. The study aimed to achieve an inclusive representation of approximately 3,000 students, with a successful response rate of 30%, yielding 900 participants.

Data for this study was gathered via an English-language electronic questionnaire, optimized for brevity and accessibility to boost participation and data quality. The questionnaire, which obtained a satisfactory Cronbach's alpha of 0.84 indicating good internal consistency, included variables such as GPA, high school grades, achievement and aptitude test scores, and demographic details like accommodation type and language proficiency. After a pilot test, which led to language simplifications based on participant feedback, the study focused on full-time undergraduate dental students enrolled from November to December 2019. Exclusions included those not enrolled during this period or unable to complete the survey. Measures were implemented to reduce biases like response fatigue and social desirability.

Ethical clearance was obtained from the Institutional Review Boards at King Saud University (E-19–4493), and Prince Sattam Bin Abdulaziz University (PSAU2020011), with all participants providing electronic informed consent in adherence to ethical standards.

IBM-SPSS version 25 was employed for statistical analysis. Descriptive statistics characterized the sample, while Pearson’s correlation analyses examined relationships between GPA and various variables. An advanced regression analysis was performed to assess the predictive value of pre-admission criteria and covariates on students' GPA. This analysis involved:

- Recategorizing some variables for a more nuanced analysis, such as living "with" or "without parents," and parental education into "more than high school" and "high school or less."
- Excluding marital status due to the homogeneity of responses.
- Assessing the regression model's fit through R-squared and adjusted R-squared values.
- Checking assumptions of linearity, normality, and homoscedasticity through diagnostic plots.
- Evaluating potential multicollinearity using variance inflation factors.

This comprehensive approach ensured a robust exploration of factors influencing academic success in the dental student cohort, providing a solid basis for interpreting the results.

## Results

### Demographic overview of participants

This study included a diverse group of 900 dental students, with detailed demographic distributions presented in Tables 1 and 2. These tables categorize both discrete and continuous variables, offering a comprehensive overview of the participant demographics.

**Table 1:** Demographic and categorical characteristics of participants

Variable		Level	N	%
**Gender**		Male	523	58.11
		Female	377	41.89
**Marital Status**		Single / Never Married	852	94.67
		Married	37	4.11
		Others	11	1.22
**Accommodation**		With their parents	697	77.44
		In a private apartment	119	13.22
		In an apartment with roommate/s	61	6.78
		In the University residence	23	2.56
**Father’s education level**		Below high school	100	11.11
		High school	179	19.89
		Diploma/Bachelor's degree	445	49.44
		Post graduate degree	176	19.56
**Mother’s education level**		Below high school	177	19.67
		High school	246	27.33
		Diploma/Bachelor's degree	400	44.44
		Post graduate degree	77	8.56
**Dental School**	**Government**	King Saud University	59	6.56
		Princess Norah Bint Abdulrahman University	92	10.22
		King Saud Bin Abdulaziz University for Health Sciences	113	12.56
		Prince Sattam Bin Abdulaziz University	152	16.89
		Al Majmaah University	125	13.89
	**Private**	Riyadh Elm University	253	28.11
		Dar Al Uloom University	40	4.44
		Al Farabi Colleges	66	7.33
**The current academic year of the participant**		1st year	76	8.44
		2nd year	114	12.67
		3rd year	157	17.44
		4th year	195	21.67
		5th year	184	20.44
		Intern	174	19.33
**The times suspended**		None	863	95.89
		1 time	26	2.89
		More than 2 times	11	1.22
**English Proficiency**		Below Average	57	6.33
		Average	587	65.22
		Above Average	256	28.44

**Table 2:** Continuous academic and performance variables of participants

Score Questions	N	Mean	Std. deviation	Min. values	Max. values
Age	900	22.68	1.914	19	31
GPA	900	4.1881	0.60743	0.00	5
High School Grade	900	96.96	2.765	80	100
Achievement Test Score	900	78.09	8.063	57	100
Aptitude Test Score	900	79.88	7.848	56	99

The majority of participants were male (58.11%) and single (94.67%). A significant proportion (77.44%) lived with their parents, and most parents had attained a diploma or bachelor's degree (Fathers: 49.44%, Mothers: 44.44%). The students were spread across various years of study and dental colleges in Riyadh. Notably, a vast majority (95.89%) reported no history of academic suspension, with only a small fraction experiencing suspensions once (2.89%) or more than once (1.22%). English proficiency predominantly fell into the 'average' category (65.22%), with fewer students rating it as 'above average' (28.44%) or 'below average' (6.33%).

The mean age of participants was 22.68 years (SD ± 1.91). Academic performance, as measured by GPA and pre-admission test scores, is also summarized: the average GPA stood at 4.18 (SD ± 0.60), the mean high school grade was 96.96 (SD ± 2.76), and average scores for aptitude and achievement tests were 79.88 (SD ± 8.06) and 78.09 (SD ± 7.84) respectively.

Table 1 illustrates the frequency distribution of discrete variables such as gender, marital status, accommodation, parental education, dental school, year of study, suspension history, and English language proficiency. Table 2 provides a breakdown of continuous variables, including age, GPA, high school grade, and pre-admission test scores, detailing mean values, standard deviations, and range.

### Correlation analysis

Building on the demographic overview, we delved into analyzing the relationships between various academic and demographic factors and students' GPA using Pearson’s correlation coefficients. This analysis is comprehensively presented in Table 3. We observed a moderate positive correlation between high school grades and GPA (*r* = 0.328, *p* < 0.01), reinforcing the notion that higher academic achievements in high school are indicative of better performance in dental school.

**Table 3:** Pearson correlation coefficients between variables

	Current GPA	High School Grade	Achievement Test Score	Aptitude Test Score	Age	The current academic year of the participant	The times suspended
High School Grade	0.328^a^						
Achievement Test Score	0.175^a^	0.307^a^					
Aptitude Test Score	0.106^a^	0.315^a^	0.680^a^				
Age	-0.336^a^	-0.181^a^	-0.215^a^	-0.095^a^			
The Current academic year of the participant	-0.219^a^	-0.06	-0.232^a^	-0.081^b^	0.814^a^		
The times suspended	-0.236^a^	-0.123^a^	0.00	-0.02	0.147^a^	0.06	
English Proficiency	0.04	0.02	0.107^a^	0.181^a^	0.02	0.110^a^	-0.03

^a^Correlation is significant at the 0.01 level (2-tailed)

^b^Correlation is significant at the 0.05 level (2-tailed)

In addition, both achievement (*r* = 0.175, *p* < 0.01) and aptitude test scores (*r* = 0.106, *p* < 0.01) exhibited significant, albeit weaker, positive correlations with GPA. This pattern underscores the importance of pre-admission academic metrics, albeit suggesting that they are among several influential factors in determining success in dental school.

The correlation coefficients further unveiled some negative relationships. The number of times a student had been suspended showed an inverse relationship with GPA (*r* = -0.236, *p* < 0.01), suggesting that disciplinary issues might be a hindrance to academic achievement. Similarly, a negative correlation between the academic year of the student and GPA (*r* = -0.219, *p* < 0.01) was observed, indicating potential challenges faced by students in advanced years of their dental education.

These findings, derived from Pearson's correlation analysis, provide a nuanced understanding of the factors that correlate with academic performance in dental school. They highlight that while high school academic achievements hold considerable weight, other aspects like disciplinary history and year of study also play significant roles in shaping a student's GPA.

### Regression analysis

Following the correlation analysis, we conducted a comprehensive regression analysis to understand the predictors of GPA in dental students. The initial analysis included continuous variables like Current GPA, High School Grade, Achievement Test Score, and Aptitude Test Score, as well as transformed ordinal categorical variables.

Our analysis indicated medium strength correlations between GPA and factors such as Age, High School Grade, and Times Suspended. Notably, the correlation between Age and the current academic year was high (> 0.8), raising concerns about potential multicollinearity, but no correlation exceeded 0.85, suggesting minimal variable overlap.

We addressed multicollinearity concerns using the Variance Inflation Factor (VIF). The results indicated no severe multicollinearity issues, as all VIF values were below the threshold of 10. This led us to proceed with these variables in the regression analysis.

The regression model, as shown in Table 4, revealed that High School Grade and Achievement Test Score had significant positive impacts on GPA, while Age and Suspended status showed negative effects. Aptitude Test Score and Living Conditions displayed marginally significant effects. This model, with bootstrapped *p*-values, provided robust results against data distribution and outliers.

**Table 4:** Linear regression analysis results for GPA predictors

Predictor	Unstandardized Coefficients (B)	Standard Error	Standardized Coefficients (Beta)	t-value	Significance (*p*-value)	Variance Inflation Factor (VIF)
Constant	1.822	0.821	-	2.220	0.062	-
**High School Grade**	**0.049**	**0.007**	**0.226**	**6.959**	** < 0.001**	**1.214**
**Achievement Test Score**	**0.007**	**0.003**	**0.098**	**2.273**	**0.027**	**2.131**
Aptitude Test Score	-0.006	0.003	-0.081	-1.882	0.074	2.148
**Age**	**-0.120**	**0.019**	**-0.372**	**-6.226**	** < 0.001**	**4.108**
**Times Suspended**^a^	**-0.487**	**0.093**	**-0.158**	**-5.255**	** < 0.001**	**1.041**
Living Conditions^a^	0.092	0.047	0.063	1.964	0.077	1.185
Marital Status^a^	-0.098	0.098	-0.033	-1.000	0.422	1.218
Gender^a^	-0.005	0.038	-0.004	-0.137	0.901	1.129

Bootstrapped results are based on 1000 samples

^**a**^Variable was coded as a dummy variable for the regression analysis

The explanatory power of the model was moderate, with an R-squared value of 0.241 (adjusted R-squared = 0.231), indicating that about 24.1% of the variance in GPA could be explained by the predictors used. While this percentage may seem modest, it is significant given the complexity of factors influencing academic performance.

Subsequently, we explored non-linear relationships and interactions between predictors. However, these analyses did not substantially increase the explained variance. Consequently, we adopted a simpler model with linear linkages, adhering to the principle of parsimony.

The final model was selected using a stepwise procedure based on Akaike’s information criteria (AIC) [10], employing a backward method to control for potential suppressor effects. This model, as detailed in Tables 5 and 6, includes significant predictors and their interactions. The model's Multiple R-squared value is 0.3158, indicating that it explains approximately 31.58% of the variance in GPA scores.

**Table 5:** Main effects of the regression model

Predictor	Estimate	Std. Error	Wald Chi-Square	Sig	Sig. – Robust Estimator
Intercept	44.171	12.2880	12.921	< 0.001	0.002
Living with Parents	0.088	0.0452	3.780	0.052	0.103
**Married**	**-5.731**	**2.6749**	**4.590**	**0.032**	**0.021**
**Male**	**3.375**	**1.2554**	**7.228**	**0.007**	**0.019**
Was Suspended	-0.287	0.1587	3.280	0.070	0.095
**Age**	**-1.596**	**0.3306**	**23.292**	** < 0.001**	** < 0.001**
Father's Education	-0.101	0.0594	2.871	0.090	0.080
Mother's Education	-0.084	0.4397	0.036	0.849	0.876
Study Year	-0.044	0.2825	0.024	0.877	0.902
**High School Grade**	**-0.287**	**0.1217**	**5.552**	**0.018**	**0.027**
**Achievement Test Score**	**0.386**	**0.1612**	**5.746**	**0.017**	**0.037**
**Aptitude Test Score**	**-0.608**	**0.1446**	**17.689**	** < 0.001**	** < 0.001**
**English Proficiency**	**-0.890**	**0.3708**	**5.765**	**0.016**	**0.018**

**Table 6:** Interaction effects in the regression model

Predictor	Estimate	Std. Error	Wald Chi-Square	Sig	Sig. – Robust Estimator
Age: Achievement Test Score	-0.006	0.0032	3.388	0.066	0.107
**High School Grade: Achievement Test Score**	**-0.003**	**0.0014**	**5.306**	**0.021**	**0.037**
**Male: Achievement Test Score**	**-0.011**	**0.0048**	**5.260**	**0.022**	**0.023**
Married: Achievement Test Score	-0.030	0.0155	3.640	0.056	0.093
Study Year: Achievement Test Score	0.006	0.0036	2.296	0.130	0.141
**Age: High School Grade**	**0.012**	**0.0032**	**14.110**	** < 0.001**	**0.001**
Age: Mother's Education	0.035	0.0178	3.839	0.050	0.116
Age: Study Year	0.011	0.0065	2.778	0.096	0.108
Father's Education: Study Year	0.026	0.0140	3.515	0.061	0.056
Male: High School Grade	-0.024	0.0135	3.238	0.072	0.125
**Married: High School Grade**	**0.084**	**0.0289**	**8.492**	**0.004**	**0.002**
Male: Study Year	-0.046	0.0242	3.604	0.058	0.055
Male: Was Suspended	-0.341	0.1923	3.141	0.076	0.145
**Married: Mother's Education**	**-0.225**	**0.0966**	**5.432**	**0.020**	**0.041**
**Married: Was Suspended**	**0.878**	**0.3618**	**5.890**	**0.015**	**0.030**
Mother's Education: Study Year	-0.042	0.0219	3.611	0.057	0.116
**Achievement Test Score: Aptitude Test Score**	**0.001**	**0.0003**	**6.233**	**0.013**	**0.006**
**Age: Aptitude Test Score**	**0.009**	**0.0032**	**7.185**	**0.007**	**0.039**
**Aptitude Test Score: English Proficiency**	**0.012**	**0.0046**	**6.683**	**0.010**	**0.009**
**High School Grade: Aptitude Test Score**	**0.004**	**0.0012**	**11.226**	** < 0.001**	**0.006**
**Mother's Education: Aptitude Test Score**	**-0.007**	**0.0028**	**6.710**	**0.010**	**0.015**
Study Year: Aptitude Test Score	-0.007	0.0037	3.583	0.058	0.117
Married: English Proficiency	0.243	0.1495	2.646	0.104	0.250

In this final model, variables such as Age, Marital status, High School Grade, Achievement and Aptitude Test Scores, and suspension history emerged as significant predictors, each contributing uniquely to the academic performance of dental students. The Adjusted R-squared value of 0.2876 demonstrates a moderate level of predictability. This level of predictability is notable, considering the model's complexity and the number of significant predictors involved. Additionally, the model's efficacy is supported by an F-statistic of 11.22 on 35 and 851 degrees of freedom, with a *p*-value of less than 0.001, underscoring the statistical significance of the model.

The regression model's main effects, as outlined in Table 5, highlight significant predictors contributing to variations in GPA scores among dental students. Notably, being married and gender (male) showed varying degrees of influence on GPA, with 'Married' having a notable negative effect and 'Male' a positive one. Age emerged as a significant negative predictor, indicating a decrease in GPA with increasing age. Academic variables such as High School Grade, Achievement Test Score, and Aptitude Test Score played significant roles in predicting GPA, with Achievement Test Score positively influencing GPA, while High School Grade and Aptitude Test Score showed a negative impact. English Proficiency also negatively impacted GPA scores. The significance levels and robust estimators further validate these effects.

Table 6 details the interaction effects within the regression model, underscoring how the relationships between different predictors and GPA scores vary when considered in conjunction. For instance, the interaction between Male and Achievement Test Score indicates that the effect of achievement scores on GPA differs by gender. Similarly, interactions involving Age with High School Grade and Aptitude Test Score reveal the complexity of these relationships over different age groups. Marital status, in combination with High School Grade and Mother's Education, shows a significant impact on GPA, suggesting different implications of marital status depending on educational background. The interactions involving Aptitude Test Score with Achievement Test Score and English Proficiency highlight how aptitude influences GPA in the context of other academic abilities. Each of these interactions contributes to a more detailed understanding of the factors influencing academic performance in dental students.

In our analysis, we assessed the average marginal effects of pre-admission variables on the current students' GPA. This approach allowed us to understand the average impact each predictor has on GPA scores. Our findings, as shown in Table 7, reveal that certain variables have a significant influence on student GPA. Notably, Age has a negative average marginal effect, indicating that older students tend to have lower GPAs, with an average decrease of -0.08 GPA points per year (*p* < 0.001). Conversely, High School Grade positively correlates with student performance, showing an increase of + 0.04 GPA points per grade unit (*p* < 0.001).

**Table 7:** Averaged marginal effects of pre-admission variables on student GPA scores

Factor	Average Marginal Effect	SE	z	p	95% CI Lower	95% CI Upper
**Achievement Test Score**	**0.01**	**0.00**	**2.29**	**0.022**	**0.00**	**0.01**
**Age**	**-0.08**	**0.02**	**-4.02**	** < 0.001**	**-0.12**	**-0.04**
**Aptitude Test Score**	0.00	0.00	-1.36	0.175	-0.01	0.00
English Proficiency	0.06	0.03	1.83	0.067	0.00	0.13
Father’s education level	0.00	0.02	0.08	0.938	-0.04	0.05
Mother’s education level	-0.03	0.02	-1.57	0.116	-0.08	0.01
**High School Grade**	**0.04**	**0.01**	**5.16**	** < 0.001**	**0.02**	**0.05**
Living with parents	0.09	0.05	1.90	0.057	0.00	0.18
Gender (Male)	-0.03	0.04	-0.75	0.451	-0.10	0.05
Marital Status (Married)	-0.09	0.10	-0.88	0.381	-0.29	0.11
Current Study Year	0.01	0.02	0.53	0.596	-0.03	0.06
**Suspended status (Suspended)**	**-0.45**	**0.09**	**-4.77**	** < 0.001**	**-0.63**	**-0.26**

Achievement Test Score also emerged as a significant predictor, with an average increase of + 0.01 GPA points per score unit (*p* = 0.022). Interestingly, English Proficiency demonstrated a marginally significant positive effect, with an increase of + 0.06 GPA points (*p* = 0.067).

Another notable finding is the impact of Suspended Status. Students who have been suspended exhibit a significant decrease in GPA, with an average reduction of -0.45 GPA points (*p* < 0.001). In contrast, variables such as Aptitude Test Score, Father’s and Mother’s education levels, Living Conditions (specifically, living with parents), Gender, Marital Status, and Current Study Year did not show statistically significant effects on GPA within the standard significance levels.

**Fig. 1:**
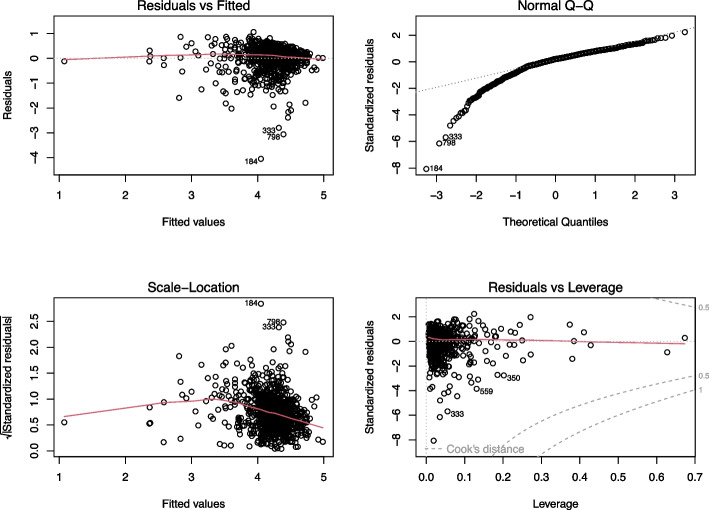
Diagnostic plots for regression model validation. **A** Residuals vs Fitted; **B** Normal Q-Q; **C** Scale-Location; **D** Residuals vs Leverage

Our regression analysis included a thorough diagnostic evaluation, as depicted in Fig. 1. This comprehensive diagnostic plot combines four key elements to assess the model's validity and robustness.

The Residuals vs Fitted Plot (Fig. 1A) demonstrated a linear relationship between the predictors and the outcome, with residuals evenly spread around a horizontal line. This suggests that the linear regression model appropriately fits the data. Additionally, the Normal Q-Q Plot (Fig. 1B) supported the normality of residuals, as indicated by the points closely aligning with the dashed line.

Concerning the assumption of homoscedasticity, the Scale-Location Plot (Fig. 1C) showed a slight deviation from the horizontal line, indicating minor heteroscedasticity. However, this issue was addressed using Huber-White sandwich estimators to produce heteroskedasticity-consistent standard errors and significance for the regression coefficients, enhancing the model's robustness.

Lastly, the Residuals vs Leverage Plot (Fig. 1D) was utilized to check for influential cases. The absence of points outside the dashed lines in this plot indicates that there are no significant influential values that could unduly affect the regression results.

Overall, these diagnostic plots collectively validate the assumptions underlying our regression model, confirming its suitability for analyzing the relationship between various predictors and student GPA.

### Additional analyses of predictor effects

#### Marital status

**Fig. 2:**
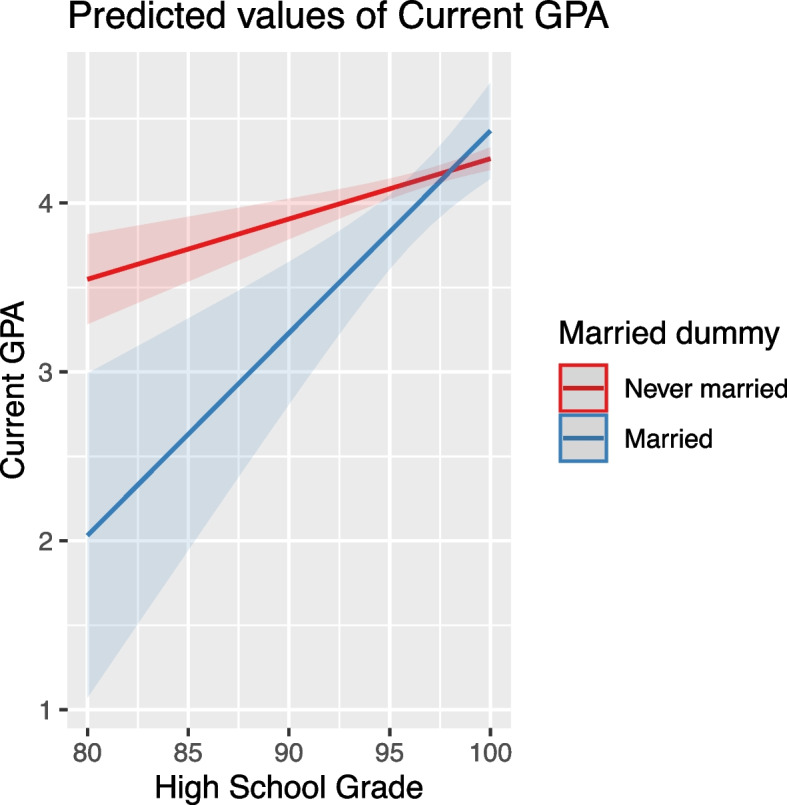
Interaction effect of marital status and high school grade on GPA

While marital status showed no significant averaged effect on student performance (Table 7), its impact varied in relation to other variables. For instance, when both high school grades and mother's education were low, married students had significantly lower GPA scores compared to their unmarried counterparts. This effect diminished with higher high school grades, suggesting an interaction effect between marital status and academic performance (Fig. 2).

#### Gender

**Fig. 3:**
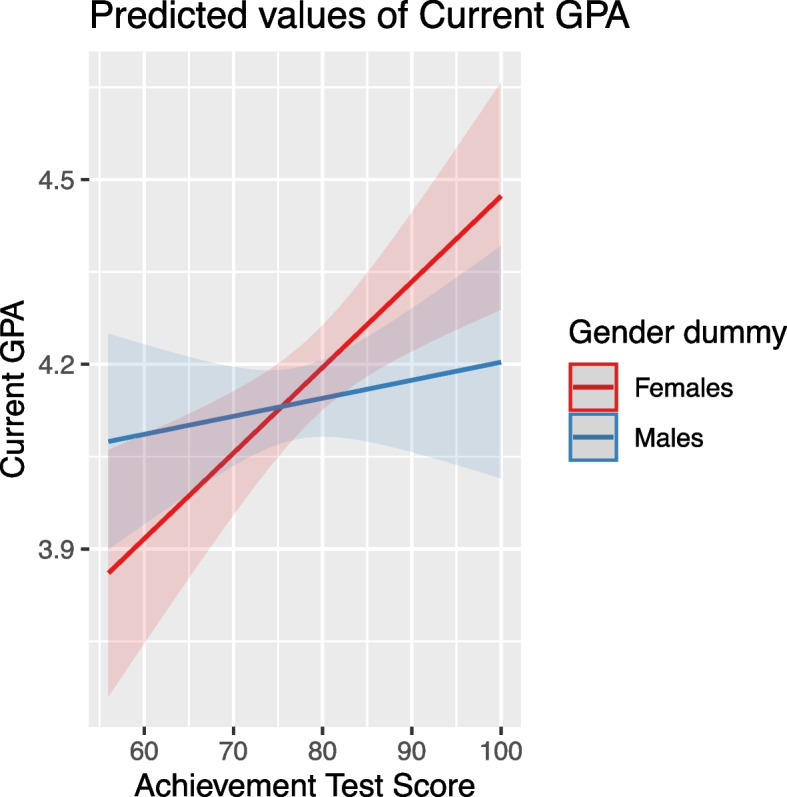
Impact of achievement test scores on GPA by gender

The average effect of gender on GPA was not significant. However, the interaction of gender with achievement test scores revealed that males outperformed females at lower achievement scores, while the opposite was true at higher scores (Fig. 3).

#### Age

**Fig. 4:**
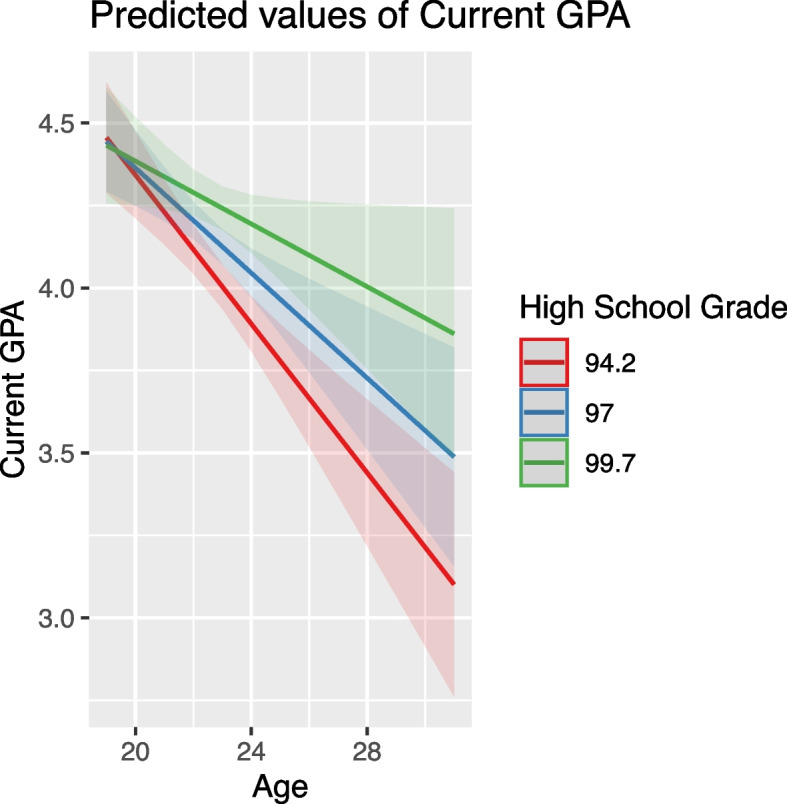
Modulating effect of high school grade on the relationship between age and GPA

**Fig. 5:**
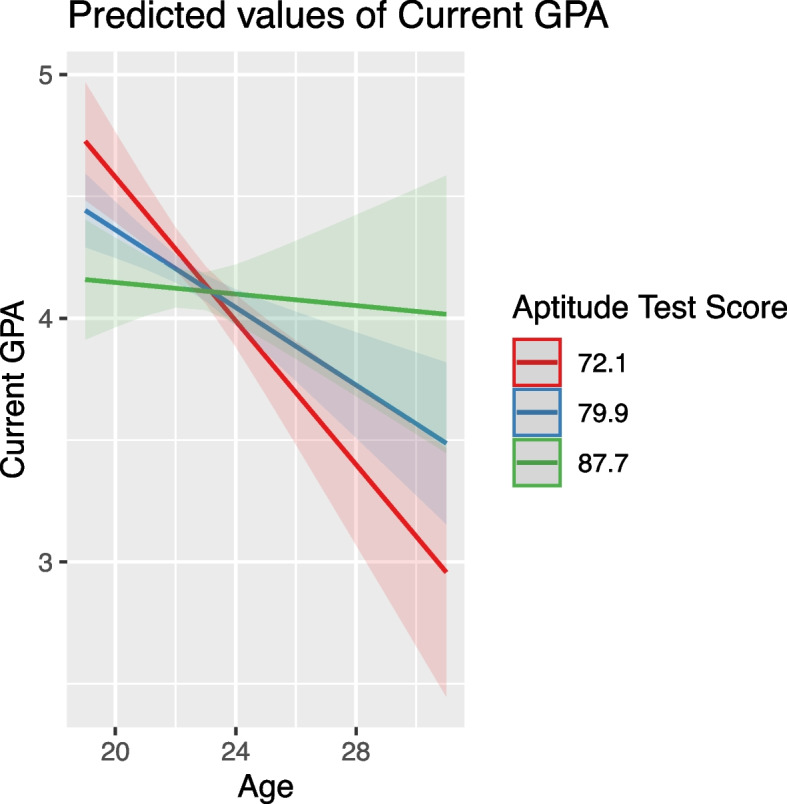
Interaction of age and aptitude test score on GPA

Age negatively impacted GPA, with older students typically having lower GPA scores. This effect was moderated by both high school grade and aptitude test score, where higher values in these predictors lessened the negative impact of age (Figs. 4 and 5).

#### High school grade

**Fig. 6:**
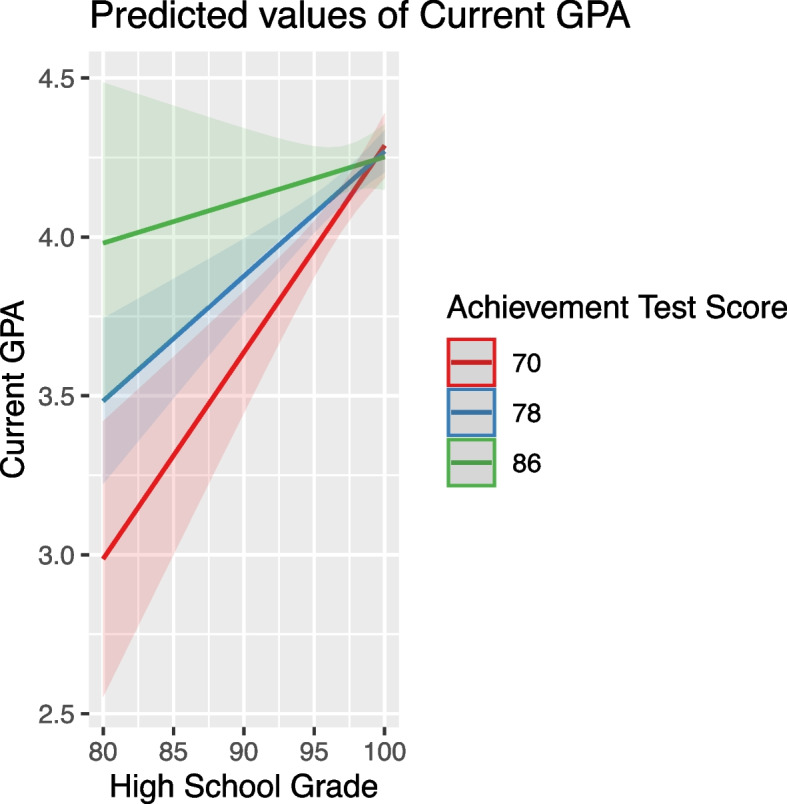
High school grade's influence on GPA in relation to achievement test scores

High school grade generally had a positive average effect on GPA, as indicated by the significant positive coefficient in Table 7. However, its impact varied under different conditions. Specifically, the interaction term High School Grade and Achievement Test Score (Fig. 6) showed that high achievement test scores reduced the positive effect of high school grades on GPA, leading to a smaller slope. This indicates that while high school grades are a positive predictor of GPA, their impact is moderated by the level of achievement test scores. Similarly, other interactions involving high school grades (as detailed in Table 6) further illustrate the nuanced nature of its impact on GPA under varying conditions.

#### Achievement and aptitude test scores

**Fig. 7:**
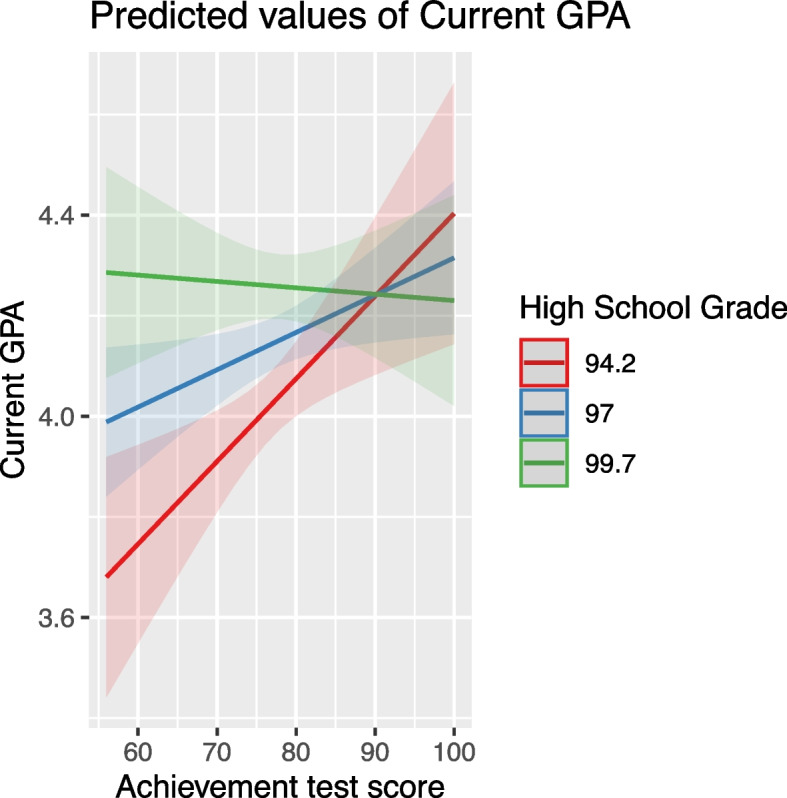
The combined effect of achievement test scores and high school grade on GPA

**Fig. 8:**
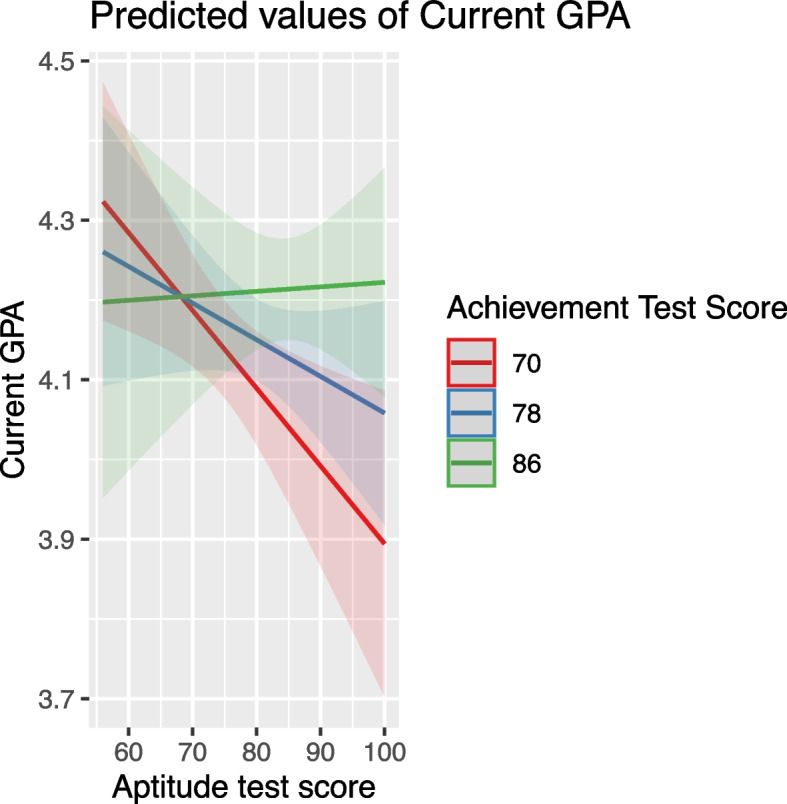
Aptitude test score's effect on GPA modified by achievement test scores

**Fig. 9:**
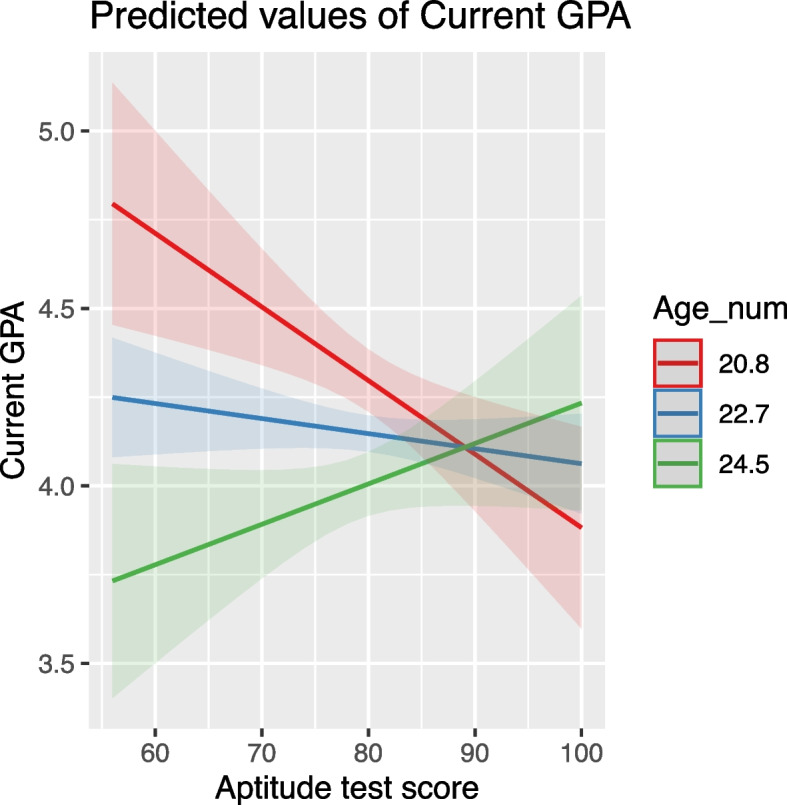
Age-dependent effect of aptitude test score on GPA

**Fig. 10:**
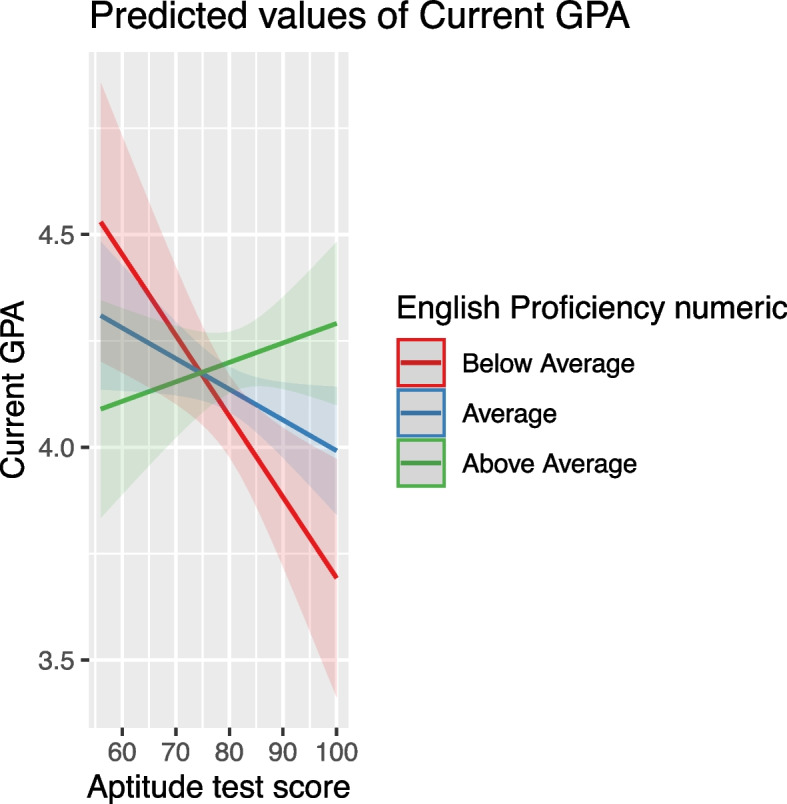
The combined effect of english proficiency and aptitude test score on GPA

These scores positively affected GPA, but their impact was moderated by other variables. For instance, high school grade influenced how achievement scores affected GPA, with the effect diminishing at higher high school grades (Fig. 7). Similarly, the effect of aptitude scores was moderated by variables like Achievement Test Scores, age, and English proficiency, indicating a nuanced relationship between these predictors and student performance (Figs. 8, 9 and 10).

#### English proficiency

**Fig. 11:**
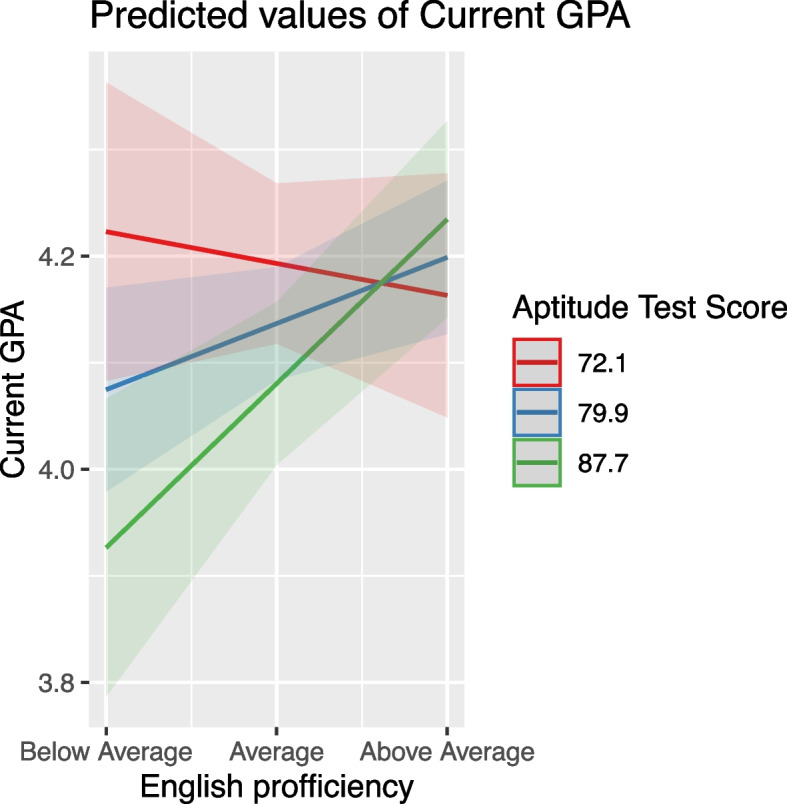
The conditional effect of english proficiency on GPA based on aptitude test scores

While the average effect of English proficiency was non-significant, it showed a significant negative impact on students' performance at low aptitude test scores. However, this effect became positive at higher aptitude test scores, demonstrating the conditional nature of English proficiency's impact (Fig. 11).

#### Suspended status

**Fig. 12:**
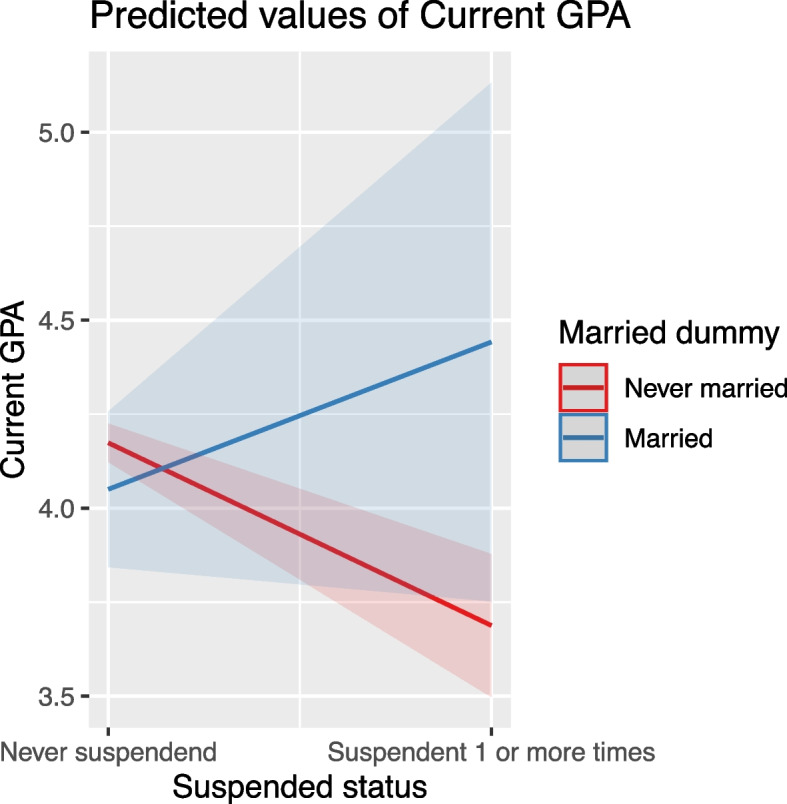
The differential impact of suspended status on GPA by marital status

Being suspended had a significantly negative effect on GPA. Interestingly, this effect turned positive for married students, indicating a unique interaction between marital and suspended statuses (Fig. 12).

#### Multilevel model analysis

Considering that data came from eight different schools, a multilevel model with random intercepts was estimated. The results were largely similar to the multiple OLS regression, with the only notable difference being the significant average marginal effect of mother's education. This consistency supports the multiple OLS regression approach, with results detailed in Tables 5, 6 and 7.

## Discussion

This study's in-depth analysis provides valuable insights into the complex factors that influence GPA among dental students in Riyadh, Saudi Arabia, highlighting the interplay of demographic, academic, and motivational elements. Key findings include moderate positive correlations of high school grades and achievement test scores with GPA, while age and suspension history showed negative impacts. The regression analysis identified significant predictors such as age, high school grades, and test scores, with interaction effects highlighting the complex influences on GPA. For example, age's negative effect on GPA was moderated by high school grade and aptitude test score. These insights demonstrate the multifaceted nature of academic performance, contributing to a more comprehensive evaluation of dental education admission strategies.

Consistent with previous research, our findings underscore the importance of high school grades as a predictor of GPA in dental school [11, 12]. The moderate correlation observed suggests that while foundational academic skills are crucial, they are part of a broader spectrum of influences on academic performance, including age and disciplinary history. Also, the roles of achievement and aptitude test scores in predicting GPA were moderated by other variables, reflecting the complex nature of academic achievement in higher education.

Age emerged as a significant predictor in our study, with older students typically demonstrating lower GPAs. This trend might be attributed to the increasing responsibilities or shifts in academic priorities that often accompany age. Adult students often juggle diverse commitments such as work, family, and community, impacting their academic engagement [13]. Additionally, life circumstances like being divorced or having young children, which are more prevalent among older students, can affect their academic performance [14]. These factors could contribute to the observed lower GPAs among older dental students, reflecting the unique challenges faced by nontraditional students in higher education. Notably, the negative effect of age on GPA appeared to be moderated by factors like high school and aptitude test scores.

In exploring gender dynamics, our findings suggest that gender alone does not have a significant average effect on GPA. However, when intersected with achievement test scores, it becomes evident that gender influences academic performance differently across various achievement levels. This aligns with previous research suggesting that gender differences in academic motivations and strategies can impact educational outcomes [15].

The study also delved into the roles of marital status in influencing GPA. While these factors alone did not show a significant average effect, their interaction with other variables like achievement test scores revealed a complex pattern of influence, underscoring the nuanced nature of these relationships. Also, the study revealed a unique role of suspended status. While being suspended generally have a negative impact on GPA, this effect becomes positive in the context of married students, suggesting an interaction between personal life circumstances and academic outcomes.

In addition, English proficiency demonstrated a conditional impact on GPA. The negative effect at lower aptitude test scores contrasted with a positive impact at higher scores, emphasizing the role of language skills in academic success, especially where English is a predominant medium of instruction.

Furthermore, our research aligns with and builds upon findings from a study on a similar group of students in the Riyadh region of Saudi Arabia [4], which examined the role of motivation in the academic performance of dental students. This study, involving a sample of undergraduate dental students, revealed positive correlations between motivation, including self-efficacy for learning and control of learning beliefs, and GPA. The negative correlation of test anxiety with GPA also emerged, highlighting the multifaceted impact of motivational factors on academic outcomes.

The limitations of the current study include its cross-sectional nature, which limits the ability to draw causal conclusions. Longitudinal studies would be beneficial for understanding how these relationships evolve throughout the educational journey. Future research might also benefit from incorporating more objective measures or triangulating data sources to enhance validity. Additionally, the study's focus on a specific geographic region may impact the generalizability of the findings. However, these insights contribute to a more comprehensive understanding of the determinants of academic success in dental education. Moreover, while the study considered key aspects, it overlooked variables such as personal motivation, teaching quality, learning environment, and mental health, all of which can significantly influence academic performance. This omission was due to concerns about creating an overly lengthy questionnaire, which could potentially reduce response rates and further complicate data analysis. Future research should include these factors for a more complete picture.

Additionally, the response rate of our study warrants consideration. We achieved a response rate of 30%, which while satisfactory, introduces certain limitations. This rate potentially influences the representativeness of our sample and the generalizability of our findings. Despite this, the diverse and substantial sample size offers valuable insights into the academic performance of dental students in Riyadh. Future research could aim for higher response rates or employ different data collection methods to enhance representativeness and further validate these findings.

Moreover, the use of an online questionnaire in this study introduces certain limitations and potential biases. While convenient, this method relies heavily on self-reported data, which can be subject to inaccuracies and personal biases. Participants may interpret questions differently, and the absence of a researcher to clarify doubts can lead to varied responses. Furthermore, the nature of online surveys may lead to selection bias, as it largely depends on the willingness and internet accessibility of participants. These factors should be considered when interpreting the study's findings, and future research might explore additional data collection methods to address these limitations.

## Conclusion

This study provides valuable insights into the factors affecting academic performance among dental students. It highlights the complexity of predicting academic success, underscoring the interactions between various demographic and academic variables. Key findings include the nuanced effects of gender, age, high school grades, and test scores on GPA, as well as the significant role of English proficiency and suspended status under specific conditions. These results contribute to a deeper understanding of academic performance in higher education and underscore the need for tailored academic support strategies that consider the diverse backgrounds and experiences of students. While the study offers important implications for educators and policymakers in dental education, its findings are also relevant to the broader discourse on academic success in higher education.

## Data Availability

The datasets used and/or analysed during the current study are available from the corresponding author on reasonable request.

## Abbreviation

GPA: Grade Point Average
